# Robust Joint Analysis with Data Fusion in Two-Stage Quantitative Trait Genome-Wide Association Studies

**DOI:** 10.1155/2013/843563

**Published:** 2013-08-12

**Authors:** Dong-Dong Pan, Wen-Jun Xiong, Ji-Yuan Zhou, Ying Pan, Guo-Li Zhou, Wing-Kam Fung

**Affiliations:** ^1^Department of Statistics, Yunnan University, Kunming 650091, China; ^2^Academy of Mathematics and Systems Science, Chinese Academy of Sciences, Beijing 100190, China; ^3^Department of Biostatistics, School of Public Health and Tropical Medicine, Southern Medical University, Guangzhou 510515, China; ^4^Department of Biology, Nanjing University, Nanjing 210093, China; ^5^College of Mathematics and Statistics, Chongqing University, Chongqing 40044, China; ^6^Department of Statistics and Actuarial Science, University of Hong Kong, Hong Kong

## Abstract

Genome-wide association studies (GWASs) in identifying the disease-associated genetic variants have been proved to be a great pioneering work. Two-stage design and analysis are often adopted in GWASs. Considering the genetic model uncertainty, many robust procedures have been proposed and applied in GWASs. However, the existing approaches mostly focused on binary traits, and few work has been done on continuous (quantitative) traits, since the statistical significance of these robust tests is difficult to calculate. In this paper, we develop a powerful *F*-statistic-based robust joint analysis method for quantitative traits using the combined raw data from both stages in the framework of two-staged GWASs. Explicit expressions are obtained to calculate the statistical significance and power. We show using simulations that the proposed method is substantially more robust than the *F*-test based on the additive model when the underlying genetic model is unknown. An example for rheumatic arthritis (RA) is used for illustration.

## 1. Introduction

Genome-wide association studies (GWASs) have identified a large number of genomic regions (especially single-nucleotide polymorphisms (SNPs)) with a wide variety of complex traits/diseases. In a GWAS, two most common types of data, qualitative (or binary) and quantitative (or continuous) traits, are analyzed and two contentious points are often faced; one is how to construct the test statistic considering the genetic model uncertainty and the other is how to evaluate the statistical significance for controlling the false positive rates efficiently (e.g., [1, 2]). Considering these issues, a lot of work has been done on the binary trait in the past 10 years (e.g., [3–7]). Computer algorithms have also been developed to calculated the significance level of robust tests in GWASs, taking into account the genetic model uncertainty [8]. However, few work has been done on continuous traits, only recently So and Sham [9] proposed a MAX3 based on score test statistics, and Li et al. [10] gave a MAX3 based on *F*-test statistics. Note that these tests just focus on single-marker analysis in one-stage analysis.

Although the costs of whole-genome genotyping are decreasing with the high-throughput biological technology, the total costs for a GWAS are still very expensive due to the thousands of sampling units and huge amounts of single-nucleotide polymorphisms. In order to save the costs, the two-stage design and the corresponding statistical analysis where all the SNPs are genotyped in Stage 1 on a portion of the samples and the promising SNPs with small *P*-values (e.g., <0.001) based on some efficient tests are further screened on the remaining subjects, are often adopted in practice (e.g., [11–15]).

In genetic association studies, especially GWASs, genetic markers are routinely tested under the assumption of additive effects. Although convenient to use, those tests are optimal only when the true underlying genetic model is additive so that they are not robust against the genetic model misspecification. To our best knowledge, few work has been done on the two-stage joint analysis for quantitative trait GWASs allowing for genetic model uncertainty. Here, we attempt to develop a joint analysis method with data fusion in the two-stage design using *F*-statistic, since *F*-test is commonly employed from the linear regression model for quantitative trait, and Li et al. [10] show that MAX3 based on *F*-statistics is more powerful than So and Sham's method by extensively numerical simulation.

The content of this paper is organized as follows. In Section 2, we give some notations and the proposed robust joint test statistics. Further, we derive the asymptotic distribution of the test statistics under the null and the alternative hypotheses. In Section 3, we show that the proposed joint analysis method is substantially more robust than the additive-model-based *F*-test from the numerical results of power comparison when the real genetic model is unknown. After that, an illustrative example for rheumatic arthritis (RA) is presented. Finally, we give some discussion of this paper in Section 4.

## 2. Methods

### 2.1. Notations

 Assume that *n* individuals are randomly selected to be genotyped in a two-staged GWAS for a certain quantitative trait and that *π* is the sampling proportion in Stage 1. Let *n*_1_ = *nπ* and *n*_2_ = *n*(1 − *π*) be the sample sizes for Stages 1 and 2, respectively. Consider a biallelic marker with two alleles G and g. Without loss of generality, we assume that G is the minor or high-risk allele. We suppose that the total *m* SNPs are genotyped on the samples of Stage 1, and SNPs with *P*-values less than *γ* in Stage 1 will be further genotyped and tested in Stage 2. Let the significance level be *α*, and then the genome-wide significance level per SNP is *α*/*m* with the Bonferroni adjustments. Let **Y**_1_ = (*y*_1_,*y*_2_,…,*y*_*n*_1__)′ and **Y**_2_ = (*y*_*n*_1_+1_,*y*_*n*_1_+2_,…,*y*_*n*_)′ be the observed quantitative outcome vectors for Stage 1 and Stage 2, respectively. Without loss of generality, we assume that the first *n*_10_ individuals in Stage 1 have the genotype gg, the second *n*_11_ individuals in Stage 1 have the genotype Gg, and the last *n*_12_ subjects in Stage 1 possess the genotype GG. Similarly, the first *n*_20_ subjects in Stage 2 have the genotype gg, the second *n*_21_ individuals in Stage 2 have the genotype Gg, and the last *n*_22_ subjects in Stage 2 possess the genotype GG. Let 0_*k*_ = (0,0,…, 0)_*k*×1_′ and 1_*k*_ = (1,1,…, 1)_*k*×1_′, and let **O**_*k*×*j*_ be the *k* × *j* matrix with all its entries being zero and **I**_*n*_ be the *n* × *n* identity matrix.

### 2.2. *F*-Statistic-Based Robust Joint Analysis

 We firstly briefly introduce *F*-statistic-based MAX3 by Li et al. [10] just using the data from Stage 1. Consider the following linear regression model:

(1) $$\begin{array}{c} y_{i}=\beta_{0}+g_{i}\beta_{1}+\varepsilon_{i},\;\varepsilon_{i}\sim N\left(0,\sigma^{2}\right),\;\;i=1,2,\ldots ,n_{1}, \end{array}$$

where *β*_0_ is the nuisance parameter for the intercept, *β*_1_ is the parameter of interest for genetic effect, and *g*_*i*_ is the genotype value, which takes 0, 1, or 2 corresponding to the count of G at a marker locus for the *i*th subject, *i* = 1,2,…, *n*_1_. The hypotheses of interest are

(2) $$\begin{array}{c} H_{0}:\beta_{1}=0⟷H_{1}:\beta_{1}\neq 0. \end{array}$$

The variable *g*_*i*_ in the previously stated equation is coded differently for the three common genetic models. Let **X**_1*R*_ = (1_*n*_1__, **G**_1*R*_), **X**_1*A*_ = (1_*n*_1__, **G**_1*A*_), and **X**_1*D*_ = (1_*n*_1__, **G**_1*D*_) be the design matrices under three commonly used genetic models, where **G**_1*R*_ = (0_*n*_10_+*n*_11__′,1_*n*_12__′)′ corresponds to the recessive model, **G**_1*A*_ = (*g*_1_,*g*_2_,…,*g*_*n*_1__)′ corresponds to the additive model, and **G**_1*D*_ = (0_*n*_10__′,1_*n*_11_+*n*_12__′)′ is for the dominant model. Denote **X**_1_ = (1_*n*_1__, **x**_11_, **x**_12_), where **x**_11_ = (0_*n*_10__′,1_*n*_11__′,0_*n*_12__′)′ and **x**_12_ = (0_*n*_10__′,0_*n*_11__′,1_*n*_12__′)′. The modified *F*-test statistics under the recessive, additive, and dominant models for Stage 1 are given by

(3) F1R=Y1′[X1R(X1R′X1R)−1X1R′−1n1(1n1′1n1)−11n1′]Y1Y1′[In1−X1(X1′X1)−1X1′]Y1/(n1−3)=(Z1R)2RSS1/(n1−3)  ,F1A=Y1′[X1A(X1A′X1A)−1X1A′−1n1(1n1′1n1)−11n1′]Y1Y1′[In1−X1(X1′X1)−1X1′]Y1/(n1−3)=(Z1A)2RSS1/(n1−3),F1D=Y1′[X1D(X1D′X1D)−1X1D′−1n1(1n1′1n1)−11n1′]Y1Y1′[In1−X1(X1′X1)−1X1′]Y1/(n1−3)=(Z1D)2RSS1/(n1−3),

where

(4) Z1R=(n10+n11)n12n1(y−n10+n11−y−n12),Z1D=n10(n11+n12)n1(y−n10−y−n11+n12),Z1A=(n10(n11+2n12)y−n10−n11(n10−n12)y−n11  − n12(2n10+n11)y−n12) ×(n1[n10(n11+4n12)+n11n12])−1,y−n10=1n10∑j=1n10yj,y−n11=1n11∑j=n10+1n10+n11yj,y−n12=1n12∑j=n10+n11+1n1yj,y−n10+n11=1n10+n11∑j=1n10+n11yj,y−n11+n12=1n11+n12∑j=n10+1n1yj.

The robust test statistic in Stage 1 is

(5) $$\begin{array}{c} F_{1}^{\text{MAX}}=\max\left\{F_{1}^{R},F_{1}^{A},F_{1}^{D}\right\}. \end{array}$$

We now give the proposed robust joint analysis. In the framework of two-stage design GWAS of quantitative traits, the SNPs with *P*-values less than *γ* will be genotyped on the remaining *n*_2_ subjects in Stage 2. Following the previous notation for Stage 1, corresponding to the recessive, additive, and dominant models, the genotype data in Stage 2 are denoted by **G**_2*R*_ = (0_*n*_20_+*n*_21__′,1_*n*_22__′)′, **G**_2*A*_ = (*g*_*n*_1_+1_,*g*_*n*_1_+2_,…,*g*_*n*_)′, and **G**_2*D*_ = (0_*n*_20__′,1_*n*_21_+*n*_22__′)′, respectively, and the design matrices are **X**_2*R*_ = (1_*n*_2__, **G**_2*R*_), **X**_2*A*_ = (1_*n*_2__, **G**_2*A*_), and **X**_2*D*_ = (1_*n*_2__, **G**_2*D*_), respectively. Denote **X**_2_ = (1_*n*_2__, **x**_21_, **x**_22_), where **x**_21_ = (0_*n*_20__′,1_*n*_21__′,0_*n*_22__′)′ and **x**_22_ = (0_*n*_20__′,0_*n*_21__′,1_*n*_22__′)′. Then, we can obtain three modified *F*-test statistics under the recessive, additive, and dominant models for Stage 2 similarly, and denote them by *F*_2_^*R*^, *F*_2_^*A*^, and *F*_2_^*D*^. Let **Y** = (**Y**_1_′,**Y**_2_′)′, **G**_*R*_ = (**G**_1*R*_′,**G**_2*R*_′)′, **G**_*A*_ = (**G**_1*A*_′,**G**_2*A*_′)′, and **G**_*D*_ = (**G**_1*D*_′,**G**_2*D*_′)′. Denote *N*_0_ = *n*_10_ + *n*_20_, *N*_1_ = *n*_11_ + *n*_21_, and *N*_2_ = *n*_12_ + *n*_22_ for the combined sample sizes from two stages, corresponding to three genotypes. Then the proposed *F*-test statistics under three genetic models on the basis of the combined data are as follows:

(6) FJR=Y′[XR(XR′XR)−1XR′−1n(1n′1n)−11n′]YY′[In−W(W′W)−1W′]Y/(n−6)=(ZJR)2RSSJ/(n−6),FJA=Y′[XA(XA′XA)−1XA′−1n(1n′1n)−11n′]YY′[In−W(W′W)−1W′]Y/(n−6)=(ZJA)2RSSJ/(n−6),FJD=Y′[XD(XD′XD)−1XD′−1n(1n′1n)−11n′]YY′[In−W(W′W)−1W′]Y/(n−6)=(ZJD)2RSSJ/(n−6),

where **X**_*R*_ = (1_*n*_, **G**_*R*_), **X**_*A*_ = (1_*n*_, **G**_*A*_), **X**_*D*_ = (1_*n*_, **G**_*D*_), and $W=\left(\begin{array}{cc} X_{1} & O_{n_{1}\times 3}\\ O_{n_{2}\times 3} & X_{2} \end{array}\right)$,

(7) ZJR=(N0+N1)N2n(y−01−y−2),ZJD=N0(N1+N2)n(y−0−y−12),ZJA=(N0(N1+2N2)y−0−N1(N0−N2)y−1  −N2(2N0+N1)y−2) ×(n[N0(N1+4N2)+N1N2])−1,y−0=1N0(∑j=1n10yj+∑j=n1+1n1+n20yj),y−1=1N1(∑j=n10+1n10+n11yj+∑j=n1+n20+1n1+n20+n21yj),y−2=1N2(∑j=n10+n11+1n1yj+∑j=n1+n20+n21+1nyj),y−01=N0y−0+N1y−1N0+N1,y−12=N1y−1+N2y−2N1+N2.

Furthermore, we propose the joint testing statistic as

(8) $$\begin{array}{c} F_{J}^{\text{MAX}}=\max\left\{F_{J}^{R},F_{J}^{A},F_{J}^{D}\right\}. \end{array}$$

In order to calculate the power of the proposed joint analysis, we have to get the thresholds, which is determined by the significance level. Denote the threshold for choosing the promising SNPs in Stage 1 by *u*_1_, which is the solution of

(9) $$\begin{array}{c} \text{P}{\text{r}}_{H_{0}}\left(F_{1}^{\text{MAX}}>u_{1}\right)=\gamma . \end{array}$$

Since the genome-wide significance level is *α*/*m*, in order to control the false positive rate, we have

(10) $$\begin{array}{c} \text{P}{\text{r}}_{H_{0}}\left(F_{1}^{\text{MAX}}>u_{1},F_{J}^{\text{MAX}}>u_{J}\right)=\alpha /m, \end{array}$$

where *u*_*J*_ is the cut-off point for the joint statistic. Once we have *u*_1_ and *u*_*J*_, the power is calculated by

(11) $$\begin{array}{c} \text{P}{\text{r}}_{H_{1}}\left(F_{1}^{\text{MAX}}>u_{1},F_{J}^{\text{MAX}}>u_{J}\right). \end{array}$$

We now give the detail to calculate the cut-off point and power above. The left side of (10) can be further expressed as

(12) PrH0(F1MAX>u1,FJMAX>uJ)  =1−PrH0(F1MAX≤u1)−PrH0(FJMAX≤uJ)   + PrH0(F1MAX≤u1,FJMAX≤uJ).

For controlling the type I error rate and calculating the power, we need to know the distribution or the asymptotic distribution of (*Z*_1_^*R*^,*Z*_1_^*A*^,*Z*_1_^*D*^,*Z*_*J*_^*R*^,*Z*_*J*_^*A*^,*Z*_*J*_^*D*^,RSS_1_,RRS_*J*_)′ under both *H*_0_ and *H*_1_.

Note that whether *H*_0_ or *H*_1_ holds, RSS_1_ and RSS_*J*_ and (*Z*_1_^*R*^,*Z*_1_^*A*^,*Z*_1_^*D*^,*Z*_*J*_^*R*^,*Z*_*J*_^*A*^,*Z*_*J*_^*D*^)′ are mutually independent (the proof is given in Appendix A). Denote the correlation matrix of (*Z*_1_^*R*^,*Z*_1_^*A*^,*Z*_1_^*D*^)′ by **V**_1_ = (*v*_*kl*_)_3×3_, whose entries are *v*_11_ = *v*_22_ = *v*_33_ = 1, $\;\;v_{12}=v_{21}=\sqrt{n_{12}}(2n_{10}+n_{11})/\sqrt{\left(n_{10}+n_{11})[n_{10}(n_{11}+4n_{12})+n_{11}n_{12}\right]}$, $v_{13}=v_{31}=\sqrt{n_{10}n_{12}}/\sqrt{\left(n_{10}+n_{11})(n_{11}+n_{12}\right)}$, and $v_{23}=v_{32}=\sqrt{n_{10}}(2n_{12}+n_{11})/\sqrt{\left(n_{11}+n_{12})[n_{10}(n_{11}+4n_{12})+n_{11}n_{12}\right]}$, respectively. Similarly, let **V**_*J*_ = (*v*_kl_*)_3×3_ be the correlation matrix of (*Z*_*J*_^*R*^,*Z*_*J*_^*A*^,*Z*_*J*_^*D*^)′ with *v*_11_* = *v*_22_* = *v*_33_* = 1, $v_{12}^{*}=v_{21}^{*}=\sqrt{N_{2}}(2N_{0}+N_{1})/\sqrt{\left(N_{0}+N_{1})[N_{0}(N_{1}+4N_{2})+N_{1}N_{2}\right]}$, $v_{13}^{*}=v_{31}^{*}=\sqrt{N_{0}N_{2}}/\sqrt{\left(N_{0}+N_{1})(N_{1}+N_{2}\right)}$, and $v_{23}^{*}=v_{32}^{*}=\sqrt{N_{0}}(2N_{2}+N_{1})/\sqrt{\left(N_{1}+N_{2})[N_{0}(N_{1}+4N_{2})+N_{1}N_{2}\right]}$. Then, we can derive that RRS_1_/*σ* ^2^ ~ *χ*_*n*_1_−3_^2^, RSS_*J*_/*σ* ^2^ ~ *χ*_*n*−6_^2^, and

(13) $$\begin{array}{c} {{\left(Z_{1}^{R},Z_{1}^{A},Z_{1}^{D},Z_{J}^{R},Z_{J}^{A},Z_{J}^{D}\right)}^{\prime }|}_{H_{0}}\sim N_{6}\left(0_{6},\sigma^{2}\left(\begin{array}{cc} V_{1} & \rho \\ \rho^{\prime } & V_{J} \end{array}\right)\right), \end{array}$$

where **ρ** = (*ρ*_*kl*_)_3×3_ is the correlation matrix between (*Z*_1_^*R*^,*Z*_1_^*A*^,*Z*_1_^*D*^)′ and (*Z*_*J*_^*R*^,*Z*_*J*_^*A*^,*Z*_*J*_^*D*^)′, with

(14) ρ11=Corr(Z1R,ZJR)=n(n10+n11)n12n1(N0+N1)N2,ρ12Corr(Z1R,ZJA)=nn12(2n10+n11)n1(n10+n11)[N0(N1+4N2)+N1N2],ρ13=Corr(Z1R,ZJD)=nn12n10n1(n10+n11)N0(N1+N2),ρ21Corr(Z1A,ZJR)=nn12(2n10+n11)n1[n10(n11+4n12)+n11n12](N0+N1)N2,ρ22=Corr(Z1A,ZJA)=n[n10(n11+4n12)+n11n12]n1[N0(N1+4N2)+N1N2],ρ23Corr(Z1A,ZJD)=nn10(n11+2n12)n1[n10(n11+4n12)+n11n12]N0(N1+N2),ρ31=Corr(Z1D,ZJR)=nn10n12n1(n11+n12)(N0+N1)N2,ρ32=Corr(Z1D,ZJA)=nn10(n11+2n12)n1(n11+n12)[N0(N1+4N2)+N1N2],ρ33=Corr(Z1D,ZJD)=nn10(n11+n12)n1N0(N1+N2).

Under *H*_1_, for a given odds ratio OR = exp⁡(*β*_1_) for subjects with two copies of risk allele corresponding to recessive model or one copy of risk allele corresponding to additive or dominant models, we have the following:

(i) when the true genetic model is recessive,
  (15) $$\begin{array}{c} {{\left(Z_{1}^{R},Z_{1}^{A},Z_{1}^{D},Z_{J}^{R},Z_{J}^{A},Z_{J}^{D}\right)}^{\prime }|}_{H_{1}}\sim N_{6}\left(\mu^{R},\sigma^{2}\left(\begin{array}{cc} V_{1} & \rho \\ \rho^{\prime } & V_{J} \end{array}\right)\right), \end{array}$$
 where ***μ*** ^*R*^ = (*μ*_1_^*RR*^,*μ*_1_^*RA*^,*μ*_1_^*RD*^,*μ*_*J*_^*RR*^,*μ*_*J*_^*RA*^,*μ*_*J*_^*RD*^)′ with
  (16) $$\begin{array}{c} \mu_{1}^{RR}=-\sqrt{\frac{\left(n_{10}+n_{11}\right)n_{12}}{n_{1}}}\beta_{1},\\ \mu_{1}^{RA}=\frac{-n_{12}\left(2n_{10}+n_{11}\right)\beta_{1}}{\sqrt{n_{1}\left[n_{10}\left(n_{11}+4n_{12}\right)+n_{11}n_{12}\right]}},\\ \mu_{1}^{RD}=\frac{-\sqrt{n_{10}}n_{12}\beta_{1}}{\sqrt{n_{1}\left(n_{11}+n_{12}\right)}},\\ \mu_{J}^{RR}=-\sqrt{\frac{\left(N_{0}+N_{1}\right)N_{2}}{n}}\beta_{1},\\ \mu_{J}^{RA}=\frac{-N_{2}\left(2N_{0}+N_{1}\right)\beta_{1}}{\sqrt{n\left[N_{0}\left(N_{1}+4N_{2}\right)+N_{1}N_{2}\right]}},\\ \mu_{J}^{RD}=\frac{-\sqrt{N_{0}}N_{2}\beta_{1}}{\sqrt{n\left(N_{1}+N_{2}\right)}}, \end{array}$$
(ii) when the true genetic model is additive,
  (17) $$\begin{array}{c} {{\left(Z_{1}^{R},Z_{1}^{A},Z_{1}^{D},Z_{J}^{R},Z_{J}^{A},Z_{J}^{D}\right)}^{\prime }|}_{H_{1}}\sim N_{6}\left(\mu^{A},\sigma^{2}\left(\begin{array}{cc} V_{1} & \rho \\ \rho^{\prime } & V_{J} \end{array}\right)\right), \end{array}$$
 where ***μ*** ^*A*^ = (*μ*_1_^*AR*^,*μ*_1_^*AA*^,*μ*_1_^*AD*^,*μ*_*J*_^*AR*^,*μ*_*J*_^*AA*^,*μ*_*J*_^*AD*^)′ with
  (2.2) $$\begin{array}{c} \mu_{1}^{AR}=\frac{-\sqrt{n_{12}}\left(n_{11}+2n_{10}\right)\beta_{1}}{\sqrt{n_{1}\left(n_{10}+n_{11}\right)}},\\ \mu_{1}^{AA}=-\sqrt{\frac{n_{10}\left(n_{11}+4n_{12}\right)+n_{11}n_{12}}{n_{1}}}\beta_{1},\\ \mu_{1}^{AD}=\frac{-\sqrt{n_{10}}\left(n_{11}+2n_{12}\right)\beta_{1}}{\sqrt{n_{1}\left(n_{11}+n_{12}\right)}},\\ \mu_{J}^{AR}=\frac{-\sqrt{N_{2}}\left(N_{1}+2N_{0}\right)\beta_{1}}{\sqrt{n\left(N_{0}+N_{1}\right)}},\\ \mu_{J}^{AA}=-\sqrt{\frac{N_{0}\left(N_{1}+4N_{2}\right)+N_{1}N_{2}}{n}}\beta_{1},\\ \mu_{J}^{AD}=\frac{-\sqrt{N_{0}}\left(N_{1}+2N_{2}\right)\beta_{1}}{\sqrt{n\left(N_{1}+N_{2}\right)}}, \end{array}$$
(iii) when the true genetic model is dominant,
  (19) $$\begin{array}{c} {{\left(Z_{1}^{R},Z_{1}^{A},Z_{1}^{D},Z_{J}^{R},Z_{J}^{A},Z_{J}^{D}\right)}^{\prime }|}_{H_{1}}\sim N_{6}\left(\mu^{D},\sigma^{2}\left(\begin{array}{cc} V_{1} & \rho \\ \rho^{\prime } & V_{J} \end{array}\right)\right), \end{array}$$
 where ***μ*** ^*D*^ = (*μ*_1_^*DR*^,*μ*_1_^*DA*^,*μ*_1_^*DD*^,*μ*_*J*_^*DR*^,*μ*_*J*_^*DA*^,*μ*_*J*_^*DD*^)′ with
  (20) $$\begin{array}{c} \mu_{1}^{DR}=\frac{-\sqrt{n_{12}}n_{10}\beta_{1}}{\sqrt{n_{1}\left(n_{10}+n_{11}\right)}},\\ \mu_{1}^{DA}=\frac{-n_{10}\left(n_{11}+2n_{12}\right)\beta_{1}}{\sqrt{n_{1}\left[n_{10}\left(n_{11}+4n_{12}\right)+n_{11}n_{12}\right]}},\\ \mu_{1}^{DD}=-\sqrt{\frac{n_{10}\left(n_{11}+n_{12}\right)}{n_{1}}}\beta_{1},\\ \mu_{J}^{DR}=\frac{-\sqrt{N_{2}}N_{0}\beta_{1}}{\sqrt{n\left(N_{0}+N_{1}\right)}},\\ \mu_{J}^{DA}=\frac{-N_{0}\left(N_{1}+2N_{2}\right)\beta_{1}}{\sqrt{n\left[N_{0}\left(N_{1}+4N_{2}\right)+N_{1}N_{2}\right]}},\\ \mu_{J}^{DD}=-\sqrt{\frac{N_{0}\left(N_{1}+N_{2}\right)}{n}}\beta_{1}. \end{array}$$

We develop a method for simplifying the calculations of Pr_*H*_0__(*F*_1_^MAX^ ≤ *u*_1_) and Pr_*H*_0__(*F*_*J*_^MAX^ ≤ *u*_*J*_) and Pr_*H*_0__(*F*_1_^MAX^ ≤ *u*_1_, *F*_*J*_^MAX^ ≤ *u*_*J*_). The details are included in Appendix B, and the calculations of Pr_*H*_1__(*F*_1_^MAX^ ≤ *u*_1_) and Pr_*H*_1__(*F*_*J*_^MAX^ ≤ *u*_*J*_) and Pr_*H*_1__(*F*_1_^MAX^ ≤ *u*_1_, *F*_*J*_^MAX^ ≤ *u*_*J*_) are essentially similar.

## 3. Results

### 3.1. Power Comparison

We conduct simulation studies to evaluate the performance of the proposed method under three commonly used genetic models (recessive, additive, and dominant models). We mainly compare the power of two approaches; one is the proposed method in this paper, and the other is the joint analysis based on the *F*-test statistics *F*_1_^*A*^ and *F*_*J*_^*A*^. For convenience, we refer to the proposed method as MAXFJ and AFJ for the other one. We choose the sample size *n* = 2000, and *m* = 5 × 10^5^. The proportion of subjects genotyped in Stage 1 has three levels *π* = 0.3,0.4,0.5. We set the genome-wide significance level as *α* = 0.05 and that the significance level per SNP as *α*/*m* = 1 × 10^−7^. In Stage 1, the *P*-value threshold for SNPs selected for followup is set to be 1 × 10^−4^ and 2 × 10^−4^. We assume that the Hardy-Weinberg equilibrium holds in the general sample population, and then there are on average *n* × (1 − MAF)^2^, 2*n* × MAF × (1 − MAF), and *n* × MAF^2^ individuals with genotype gg, Gg, and GG, respectively, where the minor allele frequency is set to be 0.15, 0.30 and 0.45. To make the power comparison more distinctly, we specify different genetic effect parameters *β*_1_ under three genetic models as follows: *β*_1_ = 0.5 for the recessive model, *β*_1_ = 0.3 for the additive model, and *β*_1_ = 0.4 for the dominant model.

The power results are displayed in Tables 1 and 2 for *γ* = 1 × 10^−4^ and *γ* = 2 × 10^−4^, respectively. They indicate that MAXFJ is more efficiency robust than AFJ across various inheritance models. As expected, AFJ is more powerful than MAXFJ under the additive model. However, MAFJ performs much more powerful than AFJ when the true genetic model is recessive. For instance, in Table 2, with *π* = 0.4 and MAF = 0.3, the powers of AFJ and MAXFJ are 0.101 and 0.529, respectively. In summary, MAXFJ is substantially more powerful than AFJ in two-staged GWAS of quantitative traits, when the model for AFJ is misspecified.

### 3.2. An Illustration Example: Rheumatoid Arthritis

**Figure 1:**
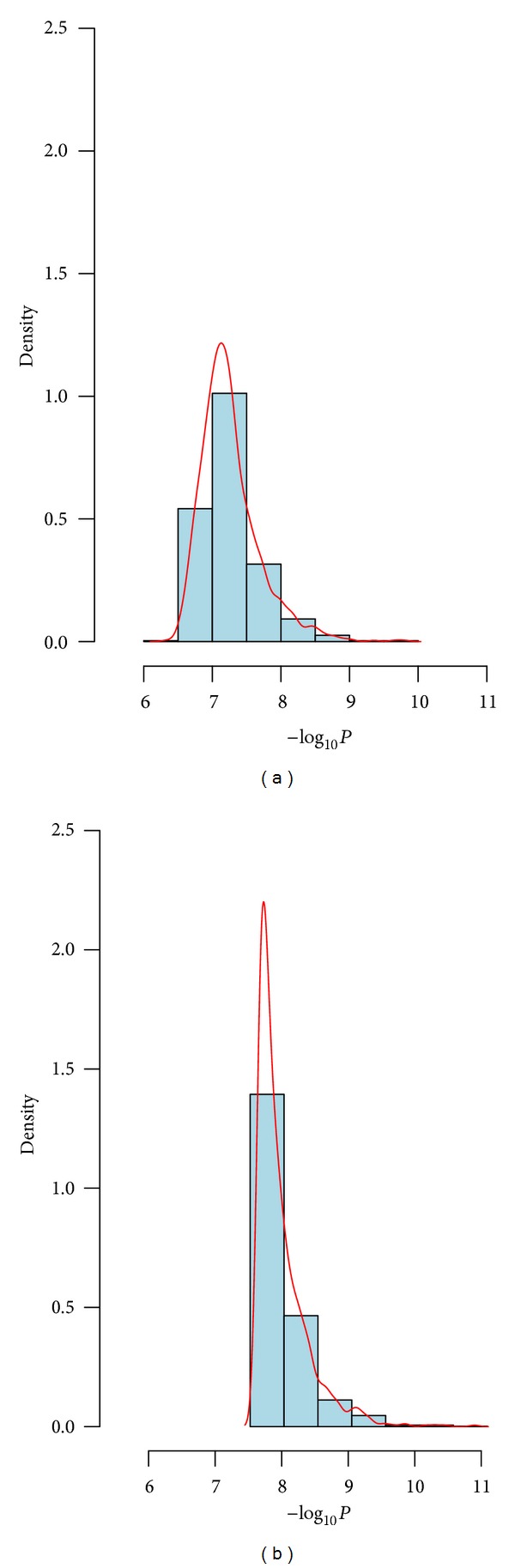
The histogram and density of −log_10_ *P* when *π* = 0.3 (the left subgraph corresponds to MAXFJ while the right one for AFJ).

**Figure 2:**
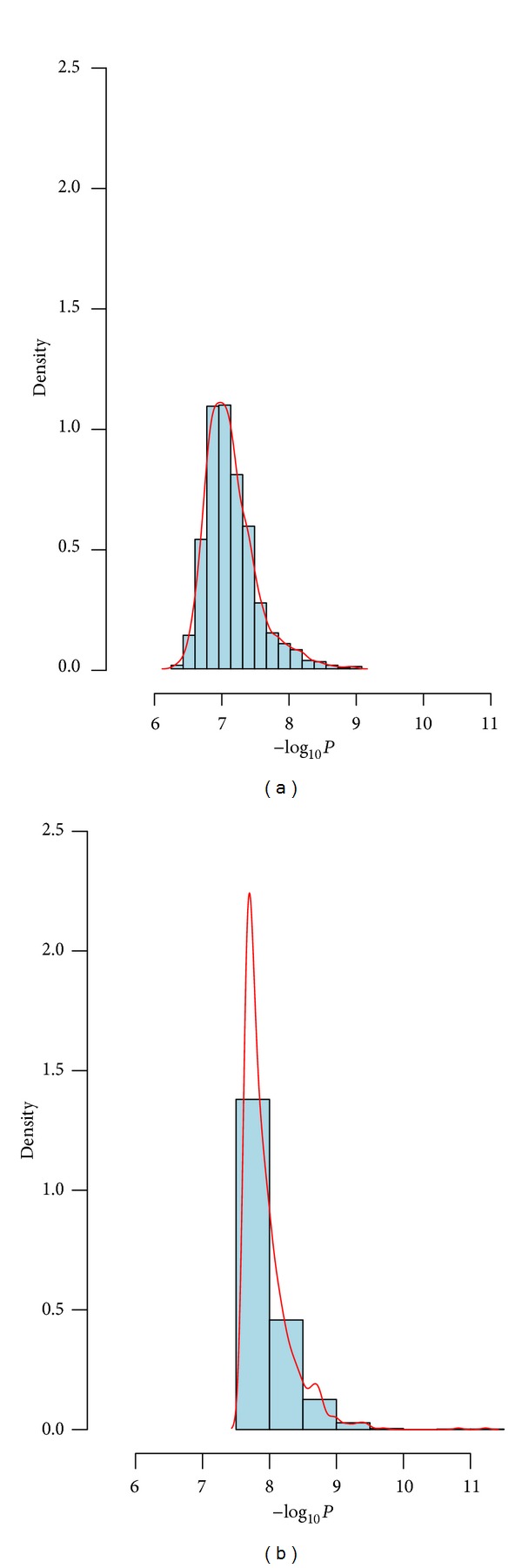
The histogram and density of −log_10_ *P* when *π* = 0.4 (the left subgraph corresponds to MAXFJ while the right one for AFJ).

**Figure 3:**
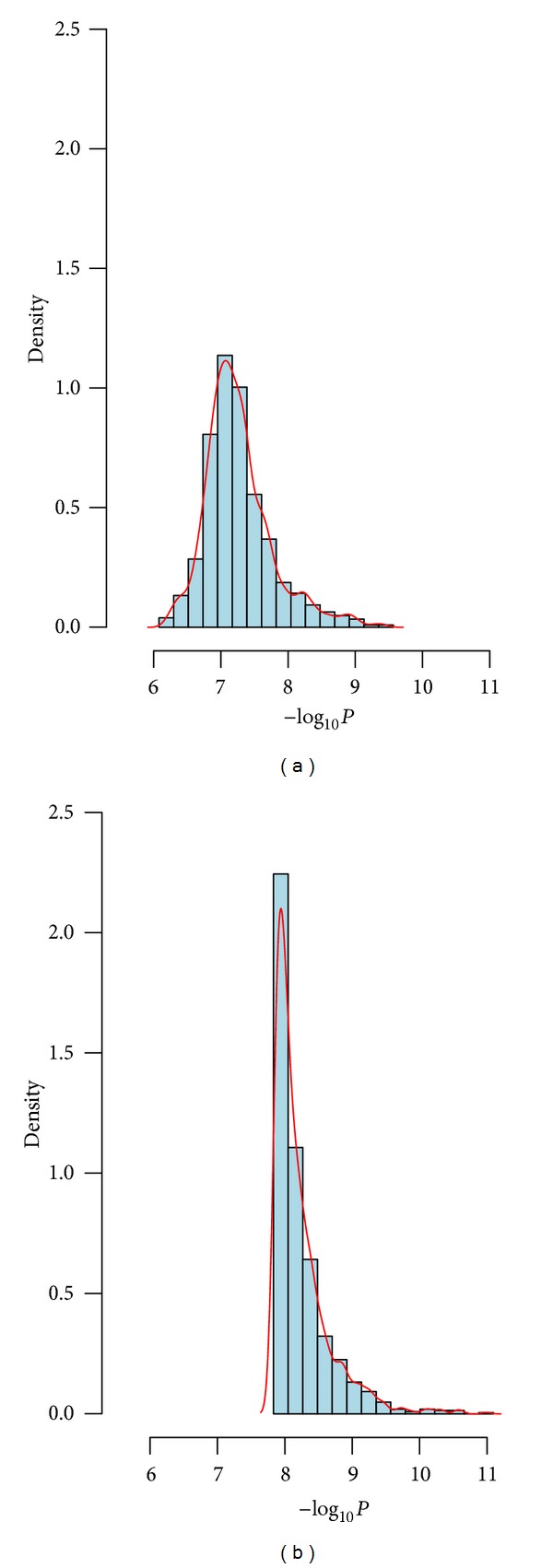
The histogram and density of −log_10_ *P* when *π* = 0.5 (the left subgraph corresponds to MAXFJ while the right one for AFJ).

Rheumatoid arthritis (RA) is an autoimmune disease (resulting in a chronically systemic inflammatory disorder) which mainly attacks synovial joints. About 1% of the common adult population worldwide is affected by RA [16]. It has been pointed out that the genetic variants might play a major role in RA susceptibility [17]. Genetic Analysis Workshop 16 (GAW16) based on the North American Rheumatoid Arthritis Consortium (NARAC) is a GWAS testing association with RA using about 5 × 10^5^ SNPs [18–20]. It included 868 individuals who were RA positive (cases) and also had continuous trait anticyclic citrullinated peptide (anti-CCP) measures and 1194 controls sampled from the New York Cancer Project (NYCP) without RA which had no anti-CCP measures. Huizinga et al. [21] pointed out that a greater anti-CCP would be linked to better prediction of increased risk developing RA. Chen et al. [22] showed that SNP rs2476601 located in PTPN22 had the most significant association with RA. Here, we only focus on SNP rs2476601 and apply two joint analysis methods (AFJ and MAXFJ) to evaluate its statistical significance. The minimum of anti-CCP among 868 cases was affected to each control, and a log transformation of anti-CCP was applied in the analysis. Then, we considered *π* = 0.3,0.4,0.5 three simulation circumstances. For *π* = 0.3, thirty percent of individuals were randomly sampled from all cases and controls and were used as the data from Stage 1, and the rest of individuals were treated as the data of Stage 2. The *P*-values of AFJ and MAXFJ were calculated, respectively. We repeated the above procedure 1,000 times and saved the corresponding *P*-values. A base-10 logarithm transformation and an opposite transformation were successively applied to these *P*-values, and the histogram and density of these transformed data were obtained (Figure 1). Similarly, we conducted the simulation and calculation for *π* = 0.4 and 0.5, and the corresponding histogram and density were presented in Figures 2 and 3. Examination of Figures 1–3 showed that the *P*-values of MAXFJ are more stable than those of AFJ and the estimated density curves of MAXFJ are more closer to the symmetrical normal distribution while the estimated density curves of AFJ are rather skewed, which indicated that MAXFJ possesses more robust performance when the real genetic models are unknown.

## 4. Discussion

We have developed a feasible two-stage design and the corresponding robust joint analysis approach for quantitative trait GWASs. The method is based on the *F*-statistics over three different genetic models. The denominator of the used *F*-statistic, which is constructed without assuming any genetic model, is different from the commonly used one. This adoption can reduce the computation intensity. Taking advantage of an ingenious design matrix, we successfully construct the common denominator of three *F*-test statistics for the joint analysis with combined raw data from both stages. The statistical significance (*P*-value) for the proposed joint analysis method can be calculated with the derived analytic expressions on the basis of the asymptotic distributions, which greatly reduce the complexity and computational intensity compared with the resampling-type permutation and bootstrap procedures. Our numerical results demonstrate that this novel approach has the greater efficiency robustness for genetic model uncertainty than the *F*-statistic-based joint analysis which assumes the additive genetic model.

In this work, we did not investigate the power of joint analysis based on other existing robust association methods for quantitative traits such as So and Sham's method. We find that it is very difficult to extend So and Sham's method (score test-based MAX3) to two-staged GWASs with quantitative outcomes, since it is almost impossible to derive the joint distribution of score tests from two stages.

For simplicity, here we do not take into account the effects of covariates in the considered two-stage design. However, in real application, the proposed method can be easily applied to the situation including one or more covariates as shown by the original MAXF by Li et al. [10]. It is important to stress that we combine the raw data from two stages to construct the joint statistic, unlike the joint analysis for binary traits using the weighted sum of two statistics in Stages 1 and 2 [12]. Furthermore, one basic assumption in this paper is that the effect sizes of genetic variants between two stages are identical (i.e., no heterogeneity exists), which is the natural and reasonable precondition for the data fusion strategy. In addition, the population-based genetic association studies may be affected by the population stratification, and this needs future research to examine it.

## Figures and Tables

**Table 1:** Power comparison (*n* = 2000, *γ* = 1 × 10^−4^, *α* = 0.05,  and *m* = 5 × 10^5^).

*π*	MAF	REC		ADD		DOM	
		AFJ	MAXFJ	AFJ	MAXFJ	AFJ	MAXFJ
0.30	0.15	7.5*e* − 5	0.005	0.426	0.365	0.610	0.618
	0.30	0.052	0.285	0.811	0.759	0.698	0.784
	0.45	0.487	0.785	0.893	0.854	0.449	0.647

0.40	0.15	1.1*e* − 4	0.009	0.651	0.589	0.826	0.837
	0.30	0.086	0.470	0.945	0.922	0.887	0.938
	0.45	0.711	0.938	0.979	0.968	0.677	0.859

0.50	0.15	1.0*e* − 4	0.010	0.802	0.751	0.933	0.941
	0.30	0.121	0.639	0.987	0.980	0.965	0.986
	0.45	0.856	0.987	0.997	0.995	0.826	0.953

**Table 2:** Power comparison (*n* = 2000, *γ* = 2 × 10^−4^, *α* = 0.05,  and *m* = 5 × 10^5^).

*π*	MAF	REC		ADD		DOM	
		AFJ	MAXFJ	AFJ	MAXFJ	AFJ	MAXFJ
0.30	0.15	1.3*e* − 4	0.006	0.489	0.426	0.676	0.681
	0.30	0.066	0.340	0.852	0.806	0.754	0.828
	0.45	0.556	0.833	0.922	0.891	0.516	0.706

0.40	0.15	1.2*e* − 4	0.011	0.709	0.651	0.866	0.876
	0.30	0.101	0.529	0.961	0.943	0.916	0.956
	0.45	0.765	0.957	0.987	0.979	0.732	0.892

0.50	0.15	1.7*e* − 4	0.012	0.838	0.793	0.951	0.958
	0.30	0.133	0.683	0.992	0.987	0.975	0.991
	0.45	0.888	0.992	0.998	0.997	0.860	0.967

## Appendix

### A. The Derivation of the Asymptotic Properties of *F*_*J*_^*R*^, *F*_*J*_^*A*^, *F*_*J*_^*D*^

Consider the linear model for the combined raw data from Stage 1 and Stage 2 as follows:

(A.1) $$\begin{array}{c} Y=W\vartheta +\varepsilon , \end{array}$$

where **ϑ** = (*β*_0_,*ζ*_1_,*ζ*_2_,*β*_0_*,*ζ*_1_*,*ζ*_2_*)′ and **ε** ~ *N*_*n*_(0_*n*_, *σ* ^2^ **I**_*n*_).

Denote

(A.2) $$\begin{array}{c} C_{R}=\left(\begin{array}{cccccc} 1 & 0 & 0 & -1 & 0 & 0\\ 0 & 1 & 0 & 0 & -1 & 0\\ 0 & 0 & 1 & 0 & 0 & -1\\ 0 & 1 & 0 & 0 & 0 & 0 \end{array}\right),\\ C_{D}=\left(\begin{array}{cccccc} 1 & 0 & 0 & -1 & 0 & 0\\ 0 & 1 & 0 & 0 & -1 & 0\\ 0 & 0 & 1 & 0 & 0 & -1\\ 0 & 1 & -1 & 0 & 0 & 0 \end{array}\right),\\ C_{A}=\left(\begin{array}{cccccc} 1 & 0 & 0 & -1 & 0 & 0\\ 0 & 1 & 0 & 0 & -1 & 0\\ 0 & 0 & 1 & 0 & 0 & -1\\ 0 & 2 & -1 & 0 & 0 & 0 \end{array}\right),\\ C_{0}=\left(\begin{array}{cccccc} 1 & 0 & 0 & -1 & 0 & 0\\ 0 & 1 & 0 & 0 & -1 & 0\\ 0 & 0 & 1 & 0 & 0 & -1\\ 0 & 1 & 0 & 0 & 0 & 0\\ 0 & 0 & 1 & 0 & 0 & 0 \end{array}\right). \end{array}$$

Based on the design matrix

(A.3) $$\begin{array}{c} W=\left(\begin{array}{cc} X_{1} & O_{n_{1}\times 3}\\ O_{n_{2}\times 3} & X_{2} \end{array}\right) \end{array}$$

for the expanded full model above, we can get the ordinary least square estimator of **ϑ** by $\hat{\vartheta }={\left(W^{\prime }W\right)}^{-1}W^{\prime }Y$, and the residual sum of square is given by RSS_*J*_ = **Y**′[**I**_*n*_ − **W**(**W**′**W**)^−1^ **W**′]**Y**. Furthermore, we denote the residual sum of squares under the following constraints: **C**_*R*_ **ϑ** = 0_4_, **C**_*D*_ **ϑ** = 0_4_, **C**_*A*_ **ϑ** = 0_4_, and **C**_0_ **ϑ** = 0_5_, by RSS_*R*_, RSS_*D*_, RSS_*A*_, and RSS_0_, respectively. After some algebras, we can obtain

(A.4) $$\begin{array}{c} F_{J}^{R}=\frac{{\text{RSS}}_{0}-{\text{RSS}}_{R}}{{\text{RSS}}_{J}/\left(n-6\right)}=\frac{\left({\text{RSS}}_{0}-{\text{RSS}}_{J}\right)-\left({\text{RSS}}_{R}-{\text{RSS}}_{J}\right)}{{\text{RSS}}_{J}/\left(n-6\right)},\\ F_{J}^{A}=\frac{{\text{RSS}}_{0}-{\text{RSS}}_{A}}{{\text{RSS}}_{J}/\left(n-6\right)}=\frac{\left({\text{RSS}}_{0}-{\text{RSS}}_{J}\right)-\left({\text{RSS}}_{A}-{\text{RSS}}_{J}\right)}{{\text{RSS}}_{J}/\left(n-6\right)},\\ F_{J}^{D}=\frac{{\text{RSS}}_{0}-{\text{RSS}}_{D}}{{\text{RSS}}_{J}/\left(n-6\right)}=\frac{\left({\text{RSS}}_{0}-{\text{RSS}}_{J}\right)-\left({\text{RSS}}_{D}-{\text{RSS}}_{J}\right)}{{\text{RSS}}_{J}/\left(n-6\right)}. \end{array}$$

On the one hand, according to

(A.5) $$\begin{array}{c} W^{\prime }W=\left(\begin{array}{cc} X_{1}^{\prime }X_{1} & O_{3\times 3}\\ O_{3\times 3} & X_{2}^{\prime }X_{2} \end{array}\right), \end{array}$$

it follows that

(A.6) $$\begin{array}{c} {\left(W^{\prime }W\right)}^{-1}=\left(\begin{array}{cc} {\left(X_{1}^{\prime }X_{1}\right)}^{-1} & O_{3\times 3}\\ O_{3\times 3} & {\left(X_{2}^{\prime }X_{2}\right)}^{-1} \end{array}\right),\\ W{\left(W^{\prime }W\right)}^{-1}W^{\prime }=\left(\begin{array}{cc} X_{1}{\left(X_{1}^{\prime }X_{1}\right)}^{-1}X_{1}^{\prime } & O_{n_{1}\times n_{2}}\\ O_{n_{2}\times n_{1}} & X_{2}{\left(X_{2}^{\prime }X_{2}\right)}^{-1}X_{2}^{\prime } \end{array}\right). \end{array}$$

So, we can get that

(A.7) Y′[In−W(W′W)−1W′]Y=Y1′[In1−X1(X1′X1)−1X1′]Y1 +Y2′[In2−X2(X2′X2)−1X2′]Y2.

That is, RSS_*J*_ = RSS_1_ + RSS_2_. Furthermore, RSS_*J*_/(*n* − 6) is also the unbiased estimator of the variance of the residual *σ* ^2^ based on the independence and unbiasedness of RSS_1_ and RSS_2_.

On the other hand, RSS_0_ − RSS_*J*_ and RSS_*R*_ − RSS_*J*_ are both independent of RSS_*J*_, so (*Z*_*J*_^*R*^)^2^ = RSS_0_ − RSS_*R*_ = (RSS_0_ − RSS_*J*_)−(RSS_*R*_ − RSS_*J*_) is also independent of RSS_*J*_, and (RSS_0_ − RSS_*R*_)/*σ* ^2^ ~ *χ*_1_^2^, RSS_*J*_/*σ* ^2^ ~ *χ*_*n*−6_^2^. Consequently, we have *F*_*J*_^*R*^ ~ *F*_1,*n*−6_. Similarly, we can get *F*_*J*_^*A*^ ~ *F*_1,*n*−6_ and *F*_*J*_^*D*^ ~ *F*_1,*n*−6_.

### B. The Detailed Calculation of Pr_*H*_0__(*F*_1_^MAX^ ≤ *u*_1_) and Pr_*H*_0__(*F*_*J*_^MAX^ ≤ *u*_*J*_) and Pr_*H*_0__(*F*_1_^MAX^ ≤ *u*_1_, *F*_*J*_^MAX^ ≤ *u*_*J*_)

Denote $w_{0}=\sqrt{(n_{10}+n_{11})n_{12}/\left(n_{10}(n_{11}+4n_{12})+n_{11}n_{12}\right)}$ and $w_{1}=\sqrt{n_{10}(n_{11}+n_{12})/\left(n_{10}(n_{11}+4n_{12})+n_{11}n_{12}\right)}$. For a given *c* > 0,

(B.1) PrH0(|Z1Rσ|≤c,|Z1Aσ|≤c,|Z1Dσ|≤c)  =∮Ω0f(z0,z1;Σ0)dz0dz1,

where *Ω*_0_ = {(*z*_0_, *z*_1_):|*z*_0_ | ≤*c*, |*w*_0_ *z*_0_ + *w*_1_ *z*_1_ | ≤*c*, |*z*_1_ | ≤*c*} and *f*(*z*_0_, *z*_1_; Σ_0_) is the bivariate normal density function for (*Z*_1_^*R*^/*σ*,*Z*_1_^*D*^/*σ*)′ with mean 0_2_ and variance-covariance matrix $\Sigma_{0}=\left(\begin{array}{cc} 1 & v_{13}\\ v_{13} & 1 \end{array}\right)$. Taking advantage of the symmetry of bivariate normal distribution, the above twofold integration can be only calculated at the right half space of *Ω*_0_ and then multiplied by 2, which is

(B.2) ∮Ω0f(z0,z1;Σ0)dz0dz1=2[∫0c(1−w1)/w0dz0∫−ccf(z0,z1;Σ0)dz1   +∫c(1−w1)/w0cdz0∫−c(c−w0z0)/w1f(z0,z1;Σ0)dz1].

Based on the property of conditional distributions of the multivariate normal distribution, we have

(B.3) $$\begin{array}{c} f\left(z_{0},z_{1};\Sigma_{0}\right)=\phi \left(z_{0}\right)f\left(z_{1}\;|\;z_{0};v_{13}\right), \end{array}$$

where *ϕ*(*z*_0_) is the probability density function of *N*(0,1) and *f*(*z*_1_ | *z*_0_; *v*_13_) is the density function of the conditional normal distribution *N*(*v*_13_ *z*_0_, 1 − *v*_13_^2^). That is,

(B.4) $$\begin{array}{c} f\left(z_{1}\;|\;z_{0};v_{13}\right)\;=\frac{1}{\sqrt{1-v_{13}^{2}}}\phi \left(\frac{z_{1}-v_{13}z_{0}}{\sqrt{1-v_{13}^{2}}}\right). \end{array}$$

Then, it follows that

(B.5) ∫0c(1−w1)/w0dz0∫−ccf(z0,z1;Σ0)dz1  =∫0c(1−w1)/w0[Φ(c−v13z01−v132)−Φ(−c−v13z01−v132)]×ϕ(z0)dz0,∫c(1−w1)/w0cdz0∫−c(c−w0z0)/w1f(z0,z1;Σ0)dz1 =∫c(1−w1)/w0c[Φ((c−w0z0)/w1−v13z01−v132)          −Φ(−c−v13z01−v132)]ϕ(z0)dz0.

Thus,

(B.6) ∮Ω0f(z0,z1;Σ0)dz0dz1=2[∫0c(1−w1)/w0Φ(c−v13z01−v132)ϕ(z0)dz0   +∫c(1−w1)/w0cΦ((c−w0z0)/w2−v13z01−v132)×ϕ(z0)dz0   −∫0cΦ(−c−v13z01−v132)ϕ(z0)dz0].

Denote $w_{0}^{*}=\sqrt{(N_{0}+N_{1})N_{2}/\left(N_{0}(N_{1}+4N_{2})+N_{1}N_{2}\right)}$ and $w_{1}^{*}=\sqrt{N_{0}(N_{1}+N_{2})/\left(N_{0}(N_{1}+4N_{2})+N_{1}N_{2}\right)}$. For any given *c*_1_, *c*_2_ > 0,

(B.7) PrH0(|Z1Rσ|≤c1,|Z1Aσ|≤c1,|Z1Dσ|≤c1,    |ZJRσ|≤c2,|ZJAσ|≤c2,|ZJDσ|≤c2) =∮Ω1f(z0,z1,z2,z3;Σ1)dz0dz1dz2dz3,

where

(B.8) Ω1={(z0,z1,z2,z3):|z0|≤c1,|w0z0+w1z1|≤c1,  |z1|≤c1,|z2|≤c2,|w0∗z2+w1∗z3|≤c2,|z3|≤c2}

and *f*(*z*_0_, *z*_1_, *z*_2_, *z*_3_; Σ_1_) is the multivariate normal density function for (*Z*_1_^*R*^/*σ*, *Z*_1_^*D*^/*σ*, *Z*_*J*_^*R*^/*σ*, *Z*_*J*_^*D*^/*σ*)′ with mean 0_4_ and variance-covariance matrix

(B.9) $$\begin{array}{c} \Sigma_{1}=\left(\begin{array}{cccc} 1 & v_{13} & \rho_{11} & \rho_{13}\\ v_{13} & 1 & \rho_{31} & \rho_{33}\\ \rho_{11} & \rho_{31} & 1 & v_{13}^{*}\\ \rho_{13} & \rho_{33} & v_{13}^{*} & 1 \end{array}\right). \end{array}$$

Note that ∮_*Ω*_1__ *f*(*z*_0_, *z*_1_, *z*_2_, *z*_3_; Σ_1_)*dz*_0_ *dz*_1_ *dz*_2_ *dz*_3_ is the sum of nine integrations as follows:

(B.10) $$\begin{array}{c} L_{1}=\int_{-c_{1}(1-w_{1})/w_{0}}^{c_{1}(1-w_{1})/w_{0}}dz_{0}\int_{-c_{1}}^{c_{1}}dz_{1}\int_{-c_{2}\left(1-w_{1}^{*}\right)/w_{0}^{*}}^{c_{2}(1-w_{1}^{*})/w_{0}^{*}}dz_{2}\int_{-c_{2}}^{c_{2}}f\left(z_{0},z_{1},z_{2},z_{3};\Sigma_{1}\right)dz_{3},\\ L_{2}=\int_{-c_{1}(1-w_{1})/w_{0}}^{c_{1}(1-w_{1})/w_{0}}dz_{0}\int_{-c_{1}}^{c_{1}}dz_{1}\int_{-c_{2}}^{-c_{2}(1-w_{1}^{*})/w_{0}^{*}}dz_{2}\int_{-(c_{2}+w_{0}^{*}z_{2})/w_{1}^{*}}^{c_{2}}f\left(z_{0},z_{1},z_{2},z_{3};\Sigma_{1}\right)dz_{3},\\ L_{3}=\int_{-c_{1}(1-w_{1})/w_{0}}^{c_{1}(1-w_{1})/w_{0}}dz_{0}\int_{-c_{1}}^{c_{1}}dz_{1}\int_{c_{2}\left(1-w_{1}^{*}\right)/w_{0}^{*}}^{c_{2}}dz_{2}\int_{-c_{2}}^{\left(c_{2}-w_{0}^{*}z_{2}\right)/w_{1}^{*}}f\left(z_{0},z_{1},z_{2},z_{3};\Sigma_{1}\right)dz_{3},\\ L_{4}=\int_{c_{1}(1-w_{1})/w_{0}}^{c_{1}}dz_{0}\int_{-c_{1}}^{\left(c_{1}-w_{0}z_{0}\right)/w_{1}}dz_{1}\int_{-c_{2}\left(1-w_{1}^{*}\right)/w_{0}^{*}}^{c_{2}(1-w_{1}^{*})/w_{0}^{*}}dz_{2}\int_{-c_{2}}^{c_{2}}f\left(z_{0},z_{1},z_{2},z_{3};\Sigma_{1}\right)dz_{3},\\ L_{5}=\int_{c_{1}(1-w_{1})/w_{0}}^{c_{1}}dz_{0}\int_{-c_{1}}^{\left(c_{1}-w_{0}z_{0}\right)/w_{1}}dz_{1}\int_{-c_{2}}^{-c_{2}(1-w_{1}^{*})/w_{0}^{*}}dz_{2}\int_{-\left(c_{2}+w_{0}^{*}z_{2}\right)/w_{1}^{*}}^{c_{2}}f\left(z_{0},z_{1},z_{2},z_{3};\Sigma_{1}\right)dz_{3},\\ L_{6}=\int_{c_{1}(1-w_{1})/w_{0}}^{c_{1}}dz_{0}\int_{-c_{1}}^{\left(c_{1}-w_{0}z_{0}\right)/w_{1}}dz_{1}\int_{c_{2}\left(1-w_{1}^{*}\right)/w_{0}^{*}}^{c_{2}}dz_{2}\int_{-c_{2}}^{\left(c_{2}-w_{0}^{*}z_{2}\right)/w_{1}^{*}}f\left(z_{0},z_{1},z_{2},z_{3};\Sigma_{1}\right)dz_{3},\\ L_{7}=\int_{-c_{1}}^{-c_{1}(1-w_{1})/w_{0}}dz_{0}\int_{-\left(c_{1}+w_{0}z_{0}\right)/w_{1}}^{c_{1}}dz_{1}\int_{-c_{2}\left(1-w_{1}^{*}\right)/w_{0}^{*}}^{c_{2}(1-w_{1}^{*})/w_{0}^{*}}dz_{2}\int_{-c_{2}}^{c_{2}}f\left(z_{0},z_{1},z_{2},z_{3};\Sigma_{1}\right)dz_{3},\\ L_{8}=\int_{-c_{1}}^{-c_{1}(1-w_{1})/w_{0}}dz_{0}\int_{-\left(c_{1}+w_{0}z_{0}\right)/w_{1}}^{c_{1}}dz_{1}\int_{-c_{2}}^{-c_{2}(1-w_{1}^{*})/w_{0}^{*}}dz_{2}\int_{-\left(c_{2}+w_{0}^{*}z_{2}\right)/w_{1}^{*}}^{c_{2}}f\left(z_{0},z_{1},z_{2},z_{3};\Sigma_{1}\right)dz_{3},\\ L_{9}=\int_{-c_{1}}^{-c_{1}(1-w_{1})/w_{0}}dz_{0}\int_{-\left(c_{1}+w_{0}z_{0}\right)/w_{1}}^{c_{1}}dz_{1}\int_{c_{2}\left(1-w_{1}^{*}\right)/w_{0}^{*}}^{c_{2}}dz_{2}\int_{-c_{2}}^{\left(c_{2}-w_{0}^{*}z_{2}\right)/w_{1}^{*}}f\left(z_{0},z_{1},z_{2},z_{3};\Sigma_{1}\right)dz_{3}. \end{array}$$

Moreover, we can obtain that *L*_2_ = *L*_3_, *L*_4_ = *L*_7_, *L*_5_ = *L*_9_, and *L*_6_ = *L*_8_ based on the symmetry of the integration domain for (*z*_0_, *z*_1_) and (*z*_2_, *z*_3_), respectively.

We have

(B.11) f(z0,z1,z2,z3;Σ1)  =ϕ(z0)f(z1 ∣ z0;v13)   ×f(z2 ∣ z0,z1;v13,ρ11,ρ31)   ×f(z3 ∣ z0,z1,z2;v13,v13∗,ρ11,ρ13,ρ31,ρ33),

where *f*(*z*_2_ | *z*_0_, *z*_1_; *v*_13_, *ρ*_11_, *ρ*_31_) is the conditional normal density function as

(B.12) f(z2 ∣ z0,z1;v13,ρ11,ρ31) =11−((ρ112−2ρ11ρ31v13+ρ312)/(1−v132))  ×ϕ[((z2−[z0(ρ11−v13ρ31)       +z1(ρ31−ρ11v13)])/(1−v132))×(1−((ρ112+2ρ11ρ31v13+ρ312)/(1−v132)))−1],

and *f*(*z*_3_ | *z*_0_, *z*_1_, *z*_2_; *v*_13_, *v*_13_*, *ρ*_11_, *ρ*_13_, *ρ*_31_, *ρ*_33_) is the conditional normal density function given by

(B.13) f(z3 ∣ z0,z1,z2;v13,v13∗,ρ11,ρ13,ρ31,ρ33)  =11−(ρ13,ρ33,v13∗)Γ−1(ρ13,ρ33,v13∗)′   ×ϕ(z3−(ρ13,ρ33,v13∗)Γ−1(z0,z1,z2)′1−(ρ13,ρ33,v13∗)Γ−1(ρ13,ρ33,v13∗)′)

with Γ the submatrix of Σ_1_ formed by first three rows and three columns.

Denote (*σ**)^2^ = (*ρ*_13_, *ρ*_33_, *v*_13_*)Γ^−1^(*ρ*_13_,*ρ*_33_,*v*_13_*)′. Then, we have

(B.14) L1=∫−c1(1−w1)/w0c1(1−w1)/w0ϕ(z0)dz0∫−c1c111−v132ϕ(z1−v13z01−v132)dz1∫−c2(1−w1∗)/w0∗c2(1−w1∗)/w0∗[Φ((c2−(ρ13,ρ33,v13∗)Γ−1(z0,z1,z2)′)(1−(σ∗)2)−1)‍−Φ((−c2−(ρ13,ρ33,v13∗)Γ−1(z0,z1,z2)′)(1−(σ∗)2)−1)]dz2,L2=∫−c1(1−w1)/w0c1(1−w1)/w0ϕ(z0)dz0∫−c1c111−v132ϕ(z1−v13z01−v132)dz1∫−c2−c2(1−w1∗)/w0∗[Φ((c2−(ρ13,ρ33,v13∗)Γ−1(z0,z1,z2)′)(1−(σ∗)2)−1)‍−Φ((−c2+w0∗z2w1∗−(ρ13,ρ33,v13∗)Γ−1(z0,z1,z2)′)(1−(σ∗)2)−1))]dz2,L4=∫c1(1−w1)/w0c1ϕ(z0)dz0∫−c1(c1−w0z0)/w111−v132ϕ(z1−v13z01−v132)dz1∫−c2(1−w1∗)/w0∗c2(1−w1∗)/w0∗[Φ((c2−(ρ13,ρ33,v13∗)Γ−1(z0,z1,z2)′)(1−(σ∗)2)−1)−Φ((−c2−(ρ13,ρ33,v13∗)Γ−1(z0,z1,z2)′) (1−(σ∗)2)−1)]dz2,L5=∫c1(1−w1)/w0c1ϕ(z0)dz0∫−c1(c1−w0z0)/w111−v132ϕ(z1−v13z01−v132)dz1∫−c2−c2(1−w1∗)/w0∗[Φ((c2−(ρ13,ρ33,v13∗)Γ−1(z0,z1,z2)′) (1−(σ∗)2)−1)‍−Φ((−c2+w0∗z2w1∗−(ρ13,ρ33,v13∗)Γ−1(z0,z1,z2)′)(1−(σ∗)2)−1)]dz2,L6=∫c1(1−w1)/w0c1ϕ(z0)dz0∫−c1(c1−w0z0)/w111−v132ϕ(z1−v13z01−v132)dz1∫c2(1−w1∗)/w0∗c2[Φ((c2−w0∗z2w1∗−(ρ13,ρ33,v13∗)Γ−1(z0,z1,z2)′)(1−(σ∗)2)−1)‍−Φ((−c2−(ρ13,ρ33,v13∗)Γ−1(z0,z1,z2)′)(1−(σ∗)2)−1))]dz2.

Finally,

(B.15) ∮Ω1f(z0,z1,z2,z3;Σ1)dz0dz1dz2dz3  =L1+2(L2+L4+L5+L6).
